# Fortifier selection and dosage enables control of breast milk osmolarity

**DOI:** 10.1371/journal.pone.0233924

**Published:** 2020-06-01

**Authors:** Ana Herranz Barbero, Nayra Rico, Benjamí Oller-Salvia, Victoria Aldecoa-Bilbao, Laura Macías-Muñoz, Robin Wijngaard, Josep Figueras-Aloy, MªDolors Salvia-Roigés

**Affiliations:** 1 Neonatology Deparment, BCNatal-Barcelona Center for Maternal Fetal and Neonatal Medicine, Hospital Clínic—Hospital Sant Joan de Déu, University of Barcelona, Barcelona, Spain; 2 Core Laboratory, Clínic Hospital, University of Barcelona, Barcelona, Spain; 3 Institut Químic de Sarrià, Universitat Ramon Llull, Barcelona, Spain; University of Illinois, UNITED STATES

## Abstract

**Background:**

Human breast milk (BM) fortification is required to feed preterm newborns with less than 32 weeks of gestation. However, addition of fortifiers increases osmolarity and osmolarity values higher than 450 mOsm/kg may be related to gastrointestinal pathology. Hence, fortifier selection and dosage are key to achieve optimal feeding.

**Objectives:**

To compare the effect on osmolality of adding different fortifications, including recently developed formulations, to BM and to study evolution of osmolarity over time in supplemented BM.

**Methods:**

Frozen mature BM from 10 healthy mothers of premature newborns was fortified with each of the following human milk fortifiers (HMF): AlmirónFortifier^®^, NANFM85^®^, or PreNANFM85^®^. In addition, fortified BMs were modified with one of the following nutritional supplements (NS): Duocal MCT^®^, Nutricia^®^ AminoAcids Mix, or Maxijul^®^. Osmolality of BM alone, fortified and/or supplemented was measured at 1 and 22 hours after their preparation. All samples were kept at 4°C throughout the study.

**Results:**

Osmolality of BM alone was close to 300 mOsm/kg and did not change over 22 hours. When equicaloric amounts of HMF AlmirónFortifier^®^, NANFM85^®^, and PreNANFM85^®^ were added to BM, osmolality increased roughly to 480 mOsm/kg with the first two fortifiers and only to 433±6 mOsm/kg with the third one. Upon addition of any of four different NSs to BM modified with AlmirónFortifier^®^ and NANFM85^®^, osmolality reached values greater than 520 mOsm/kg, while osmolality of PreNANFM85^®^ with two out of the four NSs remained below 490 mOsm/kg. NSs supplementing carbohydrates and hydrolysed proteins resulted into a higher increase of BM osmolarity. Osmolality increased significantly with time and, after 22h, only BM modified with PreNANFM85^®^ remained below 450 mOsm/kg.

**Conclusions:**

Upon addition of the HMFs tested, BM osmolality increases significantly and keeps raising over time. All HMFs but the recently developed PreNAN FM85® at 4% exceed the AAP recommended threshold for osmolarity of 450 mOsm/kg. Addition of NSs to PreNAN FM85® at 4% significantly increases osmolality above 450 mOsm/Kg. Thus, using PreNAN FM85® at 5% may be preferable to adding nutritional supplements since nutritional recommendations by the ESPGHAN are reached with a lower increase in osmolality.

## Introduction

Postnatal growth of premature infants is key to their long-term evolution [1, 2] and human breast milk (BM) is the best food option to enable optimal growth at this stage [3–6]. Composition of BM varies depending on the gestational age at which the neonate was born as well as the post-delivery days elapsed [7, 8]. However, BM alone does not provide enough nutrients for preterm newborns born before 32 weeks of gestation [9–11]. In order to reach the nutritional recommendations published by the European Society for Paediatric Gastroenterology Hepatology and Nutrition (ESPGHAN) [12, 13] and to avoid postnatal growth failure, adding human milk fortifiers (HMF) and/or nutritional supplements (NS) to BM is required [14].

HMFs and NSs needed to reach ESPGHAN nutritional recommendations increase the osmolality of BM [9, 15–17]. Enteral administration of high osmolality fluids has been associated with gastroesophageal reflux, worse gastrointestinal tolerance, and necrotizing enterocolitis [18, 19]. Hence, the American Academy of Pediatrics (AAP) advises that osmolality for enteral nutrition should not exceed 450 mOsm/kg (400 mOsm/L) [20]. Breast milk osmolality alone is already 300±6 mOsm/kg [15, 21–23] and recent reports describe that many HMFs increase osmolality above the recommended maximum levels [22, 24, 25]. Moreover, these studies do not include NSs, which are indicated for delicate and sick neonates in addition to HMFs [26] and may further increase osmotic concentration. Manufacturers recommend adding HMFs right before feeding because osmolality of fortified BM increases over time after preparation due to the hydrolysis of carbohydrates [27]. However, in many Newborn Intensive Care Units (NICUs) such as ours (Clinic Hospital of Barcelona, Spain), BM is fortified and kept up to 22 h after preparation because of three main reasons: i. to enable the planning and organization required to take care of large numbers of neonates; ii. to minimize the risk of contamination by preparing large BM volumes that avoid excessive handling; and iii. to facilitate the preparation of volumes of BM large enough that enable weighing sufficiently precise amounts of HMF with commonly available balances.

The primary objective of this study was to identify BM formulations, suitable for premature neonates, that have an osmolality within the limits recommended by AAP. To accomplish this primary goal, we established the following secondary objectives: 1. to compare osmolality of unfortified BM with that of fortified BM after adding diverse conventional HMFs and NSs at different concentrations; 2. to test whether BM fortified with the new HMF PreNAN FM85® has an osmolality within the recommended limits at the time of administration; and 3. to analyze osmolality increase in fortified BMs over time.

## Materials and methods

For this experimental study, mature BM (more than 15 days after delivery) was collected from ten healthy mothers, older than age 18, of premature newborns < 34 weeks of gestation born in our NICU (Clinic Hospital of Barcelona, Spain) between October 2015 and March 2016. As an inclusion requirement, mothers had to have milk production 20% greater than their child's needs. Milk was collected from each mother during 3 consecutive days. A volume of 50–90 mL per expression was collected from 4 to 7 expressions (350 mL total). BM was frozen at -20°C for 2 weeks. The study was approved by the Clinical Research Ethics Committee of Clinic Hospital of Barcelona (Spain) (HCB/2016/0563) and written informed consent was obtained from every mother.

**Fig 1:**
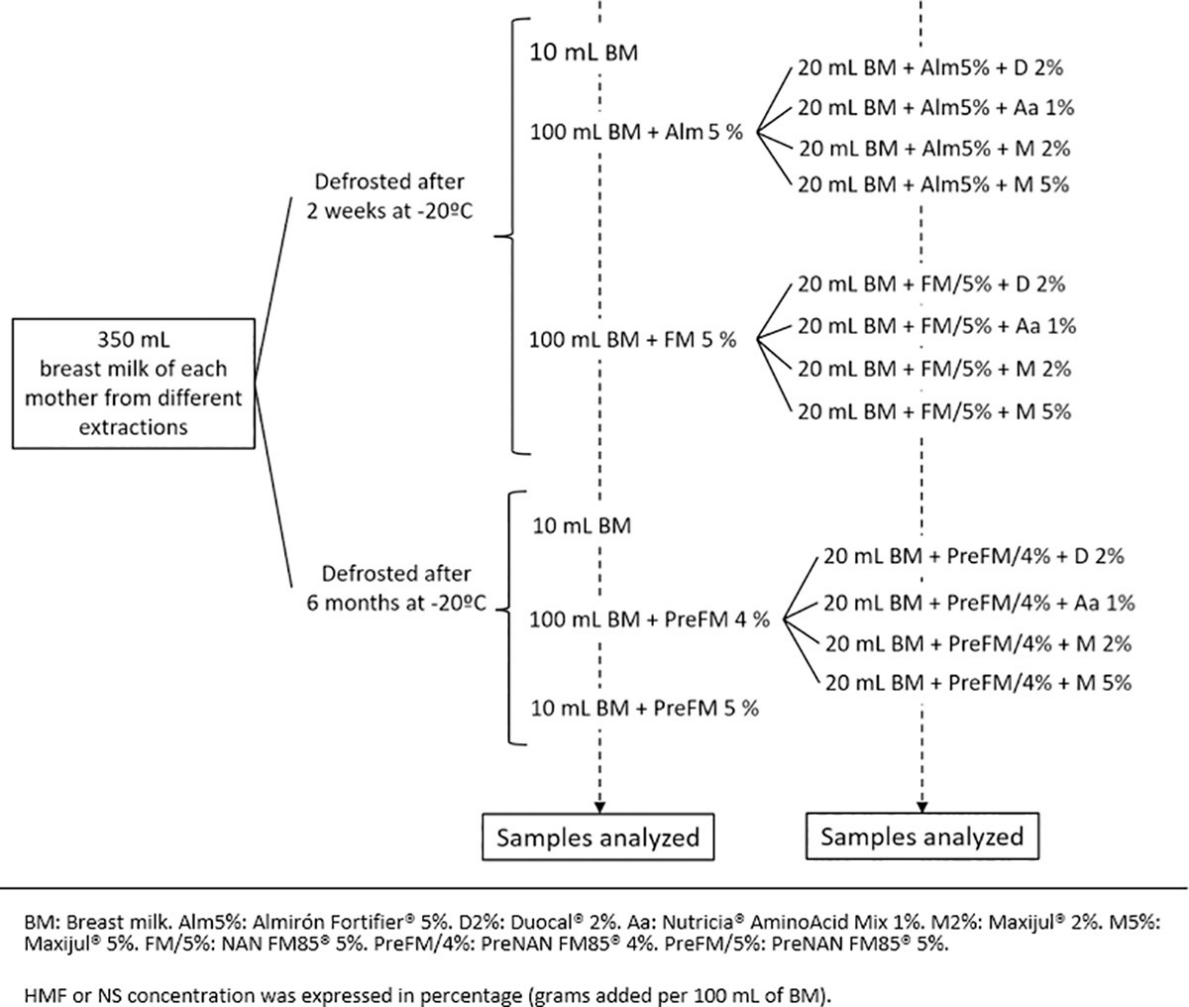
Fortifier combinations analyzed.

We tested three HMFs: Almirón Fortifier^®^ (Nutricia), NAN FM85^®^(Nestlé), and PreNAN FM85^®^(Nestlé); and three solid NSs: Duocal MCT^®^ (Nutricia), Nutricia^®^ AminoAcids Mix (Nutricia), and Maxijul^®^ (Nutricia). Each NS was added individually in addition to the HMF. PreNAN FM85^®^ was marketed after the other two other HMFs had been evaluated, so this new HMF was studied with BM from the same mothers frozen for 6 months instead of 2 weeks. HMFs were prepared at 5% (5g HMF/100mL BM). Additionally, PreNAN FM85® was tested at 4% and 5%. PreNAN FM85^®^ was tested at 5% because it increases the nutritional value of BM with a more balanced ratio of macronutrients than the one achieved by adding an NS over HMFs. Table 1 shows macronutrients contributed from each HMF and NS and Fig 1 shows the fortifiers combinations that have been analyzed.

**Table 1:** Macronutrients added for each human milk fortifier or nutritional supplement.

	Composition	Macronutrients added/gram of product			
		Kcal	Proteins	Fats	Carbohydrates
Almirón Fortifier^®^ (Nutricia)	*25% hydrolyzed protein*, *62% dextrinomaltose*	3.5	0.25	-	0.62
NAN FM85^®^ (Nestlé)	*20% hydrolyzed protein*, *66% dextrinomaltose*	3.5	0.2	0.004	0.66
PreNAN FM85^®^ (Nestlé)	*36% hydrolyzed proteins*, *32% dextrinomaltose*, *18% fats*	4.4	0.36	0.18	0.32
Duocal MCT^®^ (Nutricia)	*72*.*7% carbohydrates*, *22*.*3% fats (35% MCT)*	4.9	-	0.22	0.73
AminoAcids Mix Nutricia^®^ (Nutricia)	*98% amino acids*	3.3	0.82	-	-
Maxijul^®^ (Nutricia)	*95% carbohydrates*	3.8	-	-	0.95

The volume of BM was measured with a measuring cylinder and HMFs and NSs were weighted on weighing scales with ±0.01 g precision. Each mixture was homogenized with a magnetic stirrer (Thermolyne Nuova II) for 3 minutes. Osmolarity was analyzed with the OM-6050 Station (Menarini, Florence, Italy), which determines osmotic concentration as a function of the decrease of the freezing point. Prior to this study, we carried out the validation of the osmometer for the measurement of osmolality in BM samples. To validate this system, linearity and precision studies were performed including intra- and inter- assay analysis [28].

In order to reproduce the usual conditions of our NICU, samples were analyzed 1 h and 22 h after preparation. BM was kept at 4°C throughout the experiment. Samples were measured twice at each time point and the mean was used for the subsequent statistical analysis.

For statistical analysis SPSS (IBM SPSS Statistics Base 22.0) was used. The sample size required to detect a difference of 30 mOsm/kg (Choi et al.) [29] with an alpha risk of 0.05, a power of 80%, a standard deviation of ± 20 mOsm/kg, and assuming 20% of losses, was 10 samples per group. Mean and standard deviation of BM osmolality with and without each combination of fortifiers at the two time points were calculated. Means were compared using T-Student tests for independent or paired samples depending on the HMF used. Differences were considered statistically significant for p < 0.05. When multiple comparisons were performed to compare the HMFs NAN FM85® and PreNAN FM85®, Bonferroni post hoc analysis was used and differences were considered statistically significant for p < 0.01.

## Results

The OM-6050 Station (Menarini, Florence, Italy) displayed the precision required for this study, with an intra- and inter-assay coefficient of variation (% CV) below 2% and 1%, respectively. The regression analysis showed a linear response between 294 and 539 mOsm/kg with a coefficient of determination (R2) of 0.997 [28].

**Fig 2:**
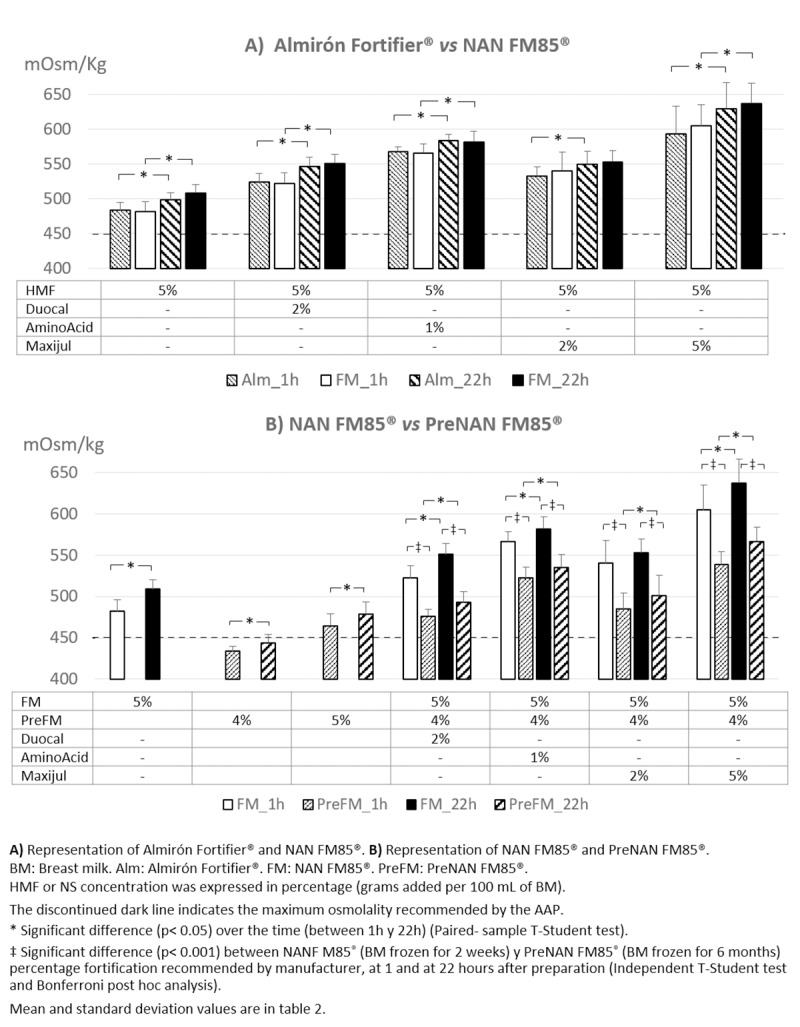
Mean osmolality of fortifier combinations, 1 and 22 hours after the preparation.

A total of 176 samples out of the 180 expected were analyzed since 4 samples were damaged before the second analysis. We first studied the effect on BM osmotic concentration of adding HMFs alone. Osmolality of BM prior to fortification was 298.7 ± 3.5 mOsm/kg. After adding the HMF Almirón Fortifier^®^ or NANFM85^®^ to BM, both prepared at 5% as instructed by the manufacturer, the osmolality increased up to 483.6 ± 10.8 mOsm/kg and 482.1 ± 14.3 mOsm/kg, respectively, 1 hour after preparation (Table 2 and Fig 2).

**Table 2:** Fortifiers combinations analyzed in the study: theoretical nutritional inputs and osmolalities measured.

					Theoretical nutritional inputs / 100mL BM without or with fortifiers				Osmolality (mOsm/kg) Mean ± ED		Osmolality increase (%)
									Time after preparation		
				n	Kcal	Proteins (g)	Carbohydrates (g)	Fat (g)	1 hour	22 hours	
Breast milk frozen for 2 weeks				10	68	1.2	7	4	298.7 ± 3.5	299.6 ± 3.4	0.3
Breast milk frozen for 6 months				10					305.4 ± 5.0 ^†^	307.8 ± 4.9 *	0.8
	BM+Alm5%			10	85.4	2.4	10.1	4	483.6 ± 10.8	498.5 ± 10.6 *	3.1
			BM+Alm5%+D2%	10	95.3	2.4	11.6	4.5	524.0 ± 12.4	546.2 ± 13.4 *	4.2
			BM+Alm5%+Aa1%	8	88.6	3.2	10.1	4	567.6 ± 7.3	583.9 ± 8.8 *	2.9
			BM+Alm5%+M2%	10	93	2.4	12	4	532.6 ± 13.5	549.6 ± 18.3 *	3.2
			BM+Alm5%+M5%	10	104.4	2.4	14.9	4	593.2 ± 39.7	629.3 ± 37.5 *	6.1
	BM+FM/5%			10	85.4	2.2	10.3	4	482.1 ± 14.3 ^‡^	508.6 ± 11.7 * ^‡^	5.5
			BM+FM/5%+D2%	10	95.3	2.2	11.8	4.5	522.3 ± 14.7 ^‡^	550.7 ± 13.3 * ^‡^	5.4
			BM+FM/5%+Aa1%	8	88.6	3	10.3	4	565.9 ± 12.6 ^‡^	581.8 ± 14.8 * ^‡^	2.8
			BM+FM/5%+M2%	10	93	2.2	12.2	4	540.2 ± 27.0 ^‡^	552.8 ± 16.6 ^‡^	2.3
			BM+FM/5%+M5%	10	104.4	2.2	15.1	4	604.4 ± 30.5 ^‡^	637.0 ± 28.7 * ^‡^	5.4
	BM+PreFM/4%			10	85.4	2.6	8.3	4.7	433.8 ± 6.1 ^‡^	444.1 ± 9.6 * ^‡^	2.4
		BM+PreFM/4%+D2%		10	95.3	2.6	9.8	5.2	476.0 ± 8.4 ^‡^	492.7 ± 13.5 * ^‡^	3.5
		BM+PreFM/4%+Aa1%		10	88.7	3.4	8.3	4.7	522.1 ± 12.9 ^‡^	535.1 ± 15.3 * ^‡^	2.5
		BM+PreFM/4%+M2%		10	93	2.6	10.2	4.7	484.6 ± 19.9 ^‡^	500.9 ± 24.4 * ^‡^	3.4
		BM+PreFM/4%+M5%		10	104.4	2.6	13	4.7	538.3 ± 15.7 ^‡^	566.5 ± 17.2 * ^‡^	5.2
	BM+PreFM/5%			10	89.8	2.9	8.6	4.9	464.0 ± 14.0	477.5 ± 15.0 *	2.9

BM: Breast milk. Alm5%: Almirón Fortifier^®^ 5%. D2%: Duocal MCT^®^ 2%. Aa: Nutricia^®^ AminoAcids Mix 1%. M2%: Maxijul^®^ 2%. M5%: Maxijul^®^ 5%. FM/5%: NAN FM85^®^ 5%. PreFM/4%: PreNAN FM85^®^ 4%. PreFM/5%: PreNAN FM85^®^ 5%.

* Significant difference (p< 0.05) over the time (between 1h and 22h) (Paired- sample T-Student test).

‡ Significant difference (p< 0.001) between NANF M85^®^ (BM frozen for 2 weeks) y PreNAN FM85^®^ (BM frozen for 6 months) percentage fortification recommended by manufacturer, at 1 and at 22 hours after preparation (Independent T-Student test and Bonferroni post hoc analysis).

† Significant difference (p< 0.05) BM without fortification after freezing for 2 weeks and 6 months (Paired- sample T-Student test).

A total of 176 samples out of the 180 expected were analyzed.

The osmolalities obtained after fortifying represent an increase of 38.0 ± 2.6 mOsm/kg and 36.6 ± 5.5 mOsm/kg per gram of added HMF, respectively (Table 3). The new HMF PreNAN FM85^®^ was prepared at 4% to match the caloric contents of the other two HMFs. Osmolarity for PreNAN FM85®- fortified BM was 433.8 ± 6.1 mOsm/kg 1 hour after preparation. The increase per gram is slightly lower for this more caloric HMF: 33.0 ± 2.4 mOsm/kg. Osmolality of the BM frozen for 6 months used for the PreNAN FM85® preparations was 305.4 ± 5.0 mOsm/kg, although it is statistically significant higher than the same BM frozen for 2 weeks (298.7 ± 3.5 mOsm/kg), a difference of 7 mOsm/kg is clinically irrelevant in this context since the variability in BM osmolality is greater than 10 mOsm/kg between different mothers and across several studies [30, 31]. Hence, results for all HMFs studied here can be compared.

**Table 3:** mOsm/kg increased per gram of HMF or NS added.

	mOsm/kg increased per gram of product added				
	1 hour after		22 hours after		Mean
Almirón Fortifier^®^	36.8	(35.7–38.0)	39.0	(37.8–40.1) *	38.0 ± 2.6
NAN FM85^®^	34.8	(32.3–37.4)	39.2	(36.1–42.2) *	36.6 ± 5.5
PreNAN FM85^®^	31.9	(31.0–32.8)	34.0	(33.0–35.1) *	33.0 ± 2.4
Duocal MCT^®^	20.4	(19.5–21.4)	23.0	(21.5-24-6) *	21.8 ± 3.6
AminoAcids Mix Nutricia^®^	87.7	(85.0–90.4)	84.8	(80.2–89.4)	86.3 ± 9.4
Maxijul^®^	24.4	(22.6–26.1)	25.4	(23.7–27.1)	24.9 ± 6.6

Values presented are mean and standar deviation or 95% confidence interval.

* Significant difference (p< 0.05) over the time (between 1h and 22h) (Paired-sample T-Student test).

After determining the effect of HMFs alone, we added each NS to BM supplemented with HMFs. Combinations of any NS with HMF Almirón Fortifier® or NANFM85® resulted into BM osmolarities between 524.0 ± 12.4 mOsm/kg and 604.4 ± 30.5 mOsm/kg one hour after preparation. When NSs where combined with PreNAN FM85® osmolality remained between 476 ± 8.4–538 ± 15.7 mOsm/Kg. The mean osmolality increase per gram of NS was: Duocal MCT^®^ 21.8 ± 3.6 mOsm/kg, Nutricia^®^ AminoAcids Mix 86.3 ± 9.4 mOsm/kg, Maxijul^®^ 24.9 ± 6.6 mOsm/kg (Table 3). The osmolality increase in BM resulting from the addition of each NS was independent of the NS-HMF combination. Osmolality increase over time was greater in combinations supplemented with carbohydrates (Maxijul^®^ and Duocal MCT^®^); the higher the concentration of supplement, the larger the increase in osmolarity.

Since NSs increased osmolality beyond recommended levels, we aimed to enhance the nutritional value of BM by increasing the HMF PreNAN FM85^®^ from 4% to 5%. Although we found this preparation increased BM osmolality up to 464 ± 14 mOsm/kg after one hour of preparation and increased over time, it remained lower than the same HMF at 4% with any NS (Table 2 and Fig 2).

Finally, we analyzed the increase in osmolality after the period of time during which fortified BM is stored in NICUs prior to feeding. After 22h at 4°C, a significant increase in osmolality was observed for all HMF and for all HMF-NS combinations, while plain BM was not significantly altered (Table 2 and Fig 2).

## Discussion

Optimizing the nutrition based on BM of premature neonates born before 32 weeks of gestation is essential to improve their growth and neurodevelopment in the medium and long term [1–6, 9–11]. In order to reach the ESPGHAN nutritional recommendations it is necessary to add HMFs and NSs to BM. However, these additives increase osmolality, which is associated with gastrointestinal complications. Here we investigate how several fortifiers modulate osmotic concentration. Upon addition of the HMFs tested, BM osmolality increases significantly and keeps raising over time. All HMFs but the recently developed PreNANFM85® at 4% exceed the recommended threshold for osmolarity of 450 mOsm/kg.

Prior to this study, we evaluated the reliability of the osmometer in the measurement of BM osmolality. A good intra- and inter-assay osmolality % CV for BM and fortified BM were obtained compared to the desirable specification of urine osmolality % CV published in the Westgard web (% CV: 14.15). Due to the similarity between the osmolality range for BM and urine, data of the urine specimen was selected in this database (Westgard QC) [32].

Because in this study we worked with frozen BM, we started by evaluating the effect of freezing on BM’s osmolality. Osmolality of plain BM after two weeks frozen at -20°C was similar to fresh BM osmolality, as previously reported [15, 21–23] (variation < 2%), and remained stable after 22 hours when BM was refrigerated at 4°C. These data are consistent with previous reports [21–23]. When frozen BM was stored for a long period of time we observed a significant increase in osmolality (< 7 mOsm/kg in 6 months), which has been reported not to be clinically relevant [30].

Subsequently, we studied the effect of HMFs on osmotic concentration. In agreement with previous publications, we observed that osmolality of BM increases significantly upon fortification and that osmolality of fortified BM increases with time unlike that of plain BM, which remains constant [22, 23, 25, 27, 29]. Lamport et al. studied two liquid HMFs and four powder infant formulas to achieve different caloric targets and they obtained osmolalities higher than 450 mOsm/kg as we have observed. Rosas et al. measured the osmolarity of NAN FM85^®^ 5% and obtained lower values than the ones reported herein: 413 ± 18 mOsm/kg vs 482.1 ± 14.3 mOsm/kg, both measured 1 h after preparation [22].

Our osmolality results are in good agreement with those obtained by Choi et al. In their study they added macronutrients separately using a targeted fortification approach [29]. They observed that osmolality of BM increases proportionally to grams of carbohydrates added in 20 mOsm/kg per gram of product. We obtained a very similar increase produced by Maxijul^®^, 24.9 ± 6.6 mOsm/kg per gram of product, since its composition is 95% carbohydrates. As could be expected, Choi et al. found that osmolality increased mostly due to the addition of hydrolyzed proteins (38 mOsm/kg) when compared to the addition of whey proteins (4 mOsm/kg). Nutricia^®^ AminoacidsMix has a high content in hydrolyzed proteins and the increase in osmolality is even higher than expected: 86.3 ± 9.4 mOsm/kg. Duocal MCT^®^ is composed 2/3 carbohydrates and 1/3 fats and thus provides the lowest osmolality increase (21.8 ± 3.6 mOsm/kg).

The AAP advises not to exceed 450 mOsm/kg in enteral nutrition [20] based on studies showing an association between high osmolality food and necrotizing enterocolitis [33, 34]. Although the threshold remains controversial [24, 25, 35, 36], many studies indicate a correlation between osmolality and gastrointestinal complications [35, 37–39]. For instance, Salvia et al. quantified reflux episodes in children from 12 months to 12 years by pH metric comparing quantity and osmolality of the food (500 mOsm/kg) concluding that osmolality delayed gastric emptying and increased the gastroesophageal reflux [37]. Moreover, Aceti et al. showed that values below 450 mOsm/kg do not result into an increase in reflux in premature newborns [38]. Nevertheless, in the last years, Miyake et al. and Lamport et al. had raised the need to reevaluate the maximum osmolality recommended by the APP [24, 25]. In our hands, BM fortified with HMF Almirón Fortifier® or NANFM85® induced osmolalites clearly over the limit recommended by AAP. Remarkably with BM fortified using equicaloric amounts of PreNAN FM85^®^, osmolality remained under 450 mOsm/Kg. To the best of our knowledge, similar studies showing the significantly lower contribution of PreNAN FM85^®^ to BM osmolality compared to other fortifiers has not yet been reported.

In order to increase the amount of nutrients supplied with BM to sick and fragile newborns, we studied combining first-line HMFs with several NSs. We found that all combinations resulted into osmolalities higher than the AAP recommended threshold, both at 1 hours and 22 hours. Hence, we envisioned an alternative way to provide higher amount of nutrients with a lower increase in osmolality. To this end, we simply increased the percentage of the new fortifier HMF PreNAN FM85^®^ from 4 to 5%. This preparation is not only more convenient than adding an NS but also contains a more balanced ratio of macronutrients. HMF PreNAN FM85^®^ 5% has an osmolality of 464.0 ± 14.0 after 1 hour and thus it approaches the AAP recommended threshold.

This experimental study has the following limitations: i. It has been performed with frozen BM, while newborns should be preferably be fed with fresh BM; although we cannot guarantee the same results after fortifying fresh BM, the change in osmolality of BM after freezing or storage has been shown to have no clinical significance [30]. ii. The first part of the experiment was performed with BM stored for 2 weeks and the second part with BM stored for 6 months. Although osmolality of the BM frozen for 6 months is statistically significant higher than the same BM frozen for 2 weeks, the difference is clinically irrelevant and results for all HMFs studied here can be compared. iii. We do not assess gastrointestinal symptoms.

Despite these limitations, all combinations of HMFs and NSs have been made with BM from the same mothers, so the differences found are directly attributable to the HMFs and NSs and not to variations in the composition of the mother's milk. We have not found other studies evaluating the osmolality of the new fortifier PreNAN FM85^®^.

## Conclusion

This study shows that BM with standard fortifiers such as HMF, NAN FM85® and Almirón Fortifier® has an osmolality much greater than 450 mOsm/Kg (477.1–498.3), which is the limit recommend by the AAP to avoid deleterious gastrointestinal effects. We have found that HMF PreNAN FM85^®^ at 4% induces an osmolality below the AAP recommended threshold even 22 hours after preparation. Upon addition of NSs to this formulation, osmolality increases well beyond 450 mOsm/Kg (530.5–564.2). Conversely, by increasing the concentration of the new fortifier from 4 to 5%, the ESPGHAN nutritional levels for weak newborns are reached, and an osmolality of 464 ± 14 mOsm/kg is achieved, which is only slightly above the recommended threshold. Overall, our study shows that using PreNAN FM85^®^ at 5% may be preferable to adding other fortifiers or nutritional supplements since ESPGHAN nutritional levels are reached with a BM osmolality close to the limit recommended by the AAP.
